# The unfolded protein response impacts melanoma progression by enhancing FGF expression and can be antagonized by a chemical chaperone

**DOI:** 10.1038/s41598-017-17888-9

**Published:** 2017-12-13

**Authors:** Karin Eigner, Yüksel Filik, Florian Mark, Birgit Schütz, Günter Klambauer, Richard Moriggl, Markus Hengstschläger, Herbert Stangl, Mario Mikula, Clemens Röhrl

**Affiliations:** 1 Center for Pathobiochemistry and Genetics, Medical University of Vienna, Vienna, Austria; 2 Institute of Bioinformatics, Johannes Kepler University, Linz, Austria; 3 Ludwig Boltzmann Institute for Cancer Research, Vienna, Austria; 4 Institute of Animal Breeding and Genetics, University of Veterinary Medicine, Vienna, Austria; 5 Medical University of Vienna, Vienna, Austria

## Abstract

The mechanisms hallmarking melanoma progression are insufficiently understood. Here we studied the impact of the unfolded protein response (UPR) - a signalling cascade playing ambiguous roles in carcinogenesis - in melanoma malignancy. We identified isogenic patient-derived melanoma cell lines harboring BRAF^V600E^-mutations as a model system to study the role of intrinsic UPR in melanoma progression. We show that the activity of the three effector pathways of the UPR (ATF6, PERK and IRE1) was increased in metastatic compared to non-metastatic cells. Increased UPR-activity was associated with increased flexibility to cope with ER stress. The activity of the ATF6- and the PERK-, but not the IRE-pathway, correlated with poor survival in melanoma patients. Using whole-genome expression analysis, we show that the UPR is an inducer of FGF1 and FGF2 expression and cell migration. Antagonization of the UPR using the chemical chaperone 4-phenylbutyric acid (4-PBA) reduced FGF expression and inhibited cell migration and viability. Consistently, FGF expression positively correlated with the activity of ATF6 and PERK in human melanomas. We conclude that chronic UPR stimulates the FGF/FGF-receptor signalling axis and promotes melanoma progression. Hence, the development of potent chemical chaperones to antagonize the UPR might be a therapeutic approach to target melanoma.

## Introduction

Melanoma is the most aggressive type of skin cancer and its incidence is rising worldwide. Once melanoma has progressed to develop distant metastasis, it is regarded as one of the most therapeutically challenging malignancies^1^. Despite recent advances in immunotherapy, no curative treatment for patients with advanced disease exists and prognosis for patients with distant metastasis is poor. Patients are confronted with a median survival time of 6–10 months only and a 3-year overall survival rate of less than 15%^2^. Therefore, there is great necessity to raise our knowledge on the processes triggering metastasis in order to improve therapies of melanoma patients with advanced stages.

Mutations in BRAF (predominantly BRAF^V600E^) are considered the main oncogenic drivers of melanoma^3^. However, the presence of a BRAF-mutation is insufficient to mediate intrinsic aggressiveness. Such mutations are present in most nevi, which persist as benign neoplasms for decades without becoming malignant. This suggests that additional features, such as the accumulation of further mutations, metabolic reprogramming and diverse cellular processes are involved in the progress of becoming a malignant disease and a metastatic tumour^4^. The unfolded protein response (UPR) might be one of these enabling characteristics involved in the development of melanoma towards metastasis, as the UPR has been identified as an ambiguous regulator of cancer progression^5^.

**Figure 1:**
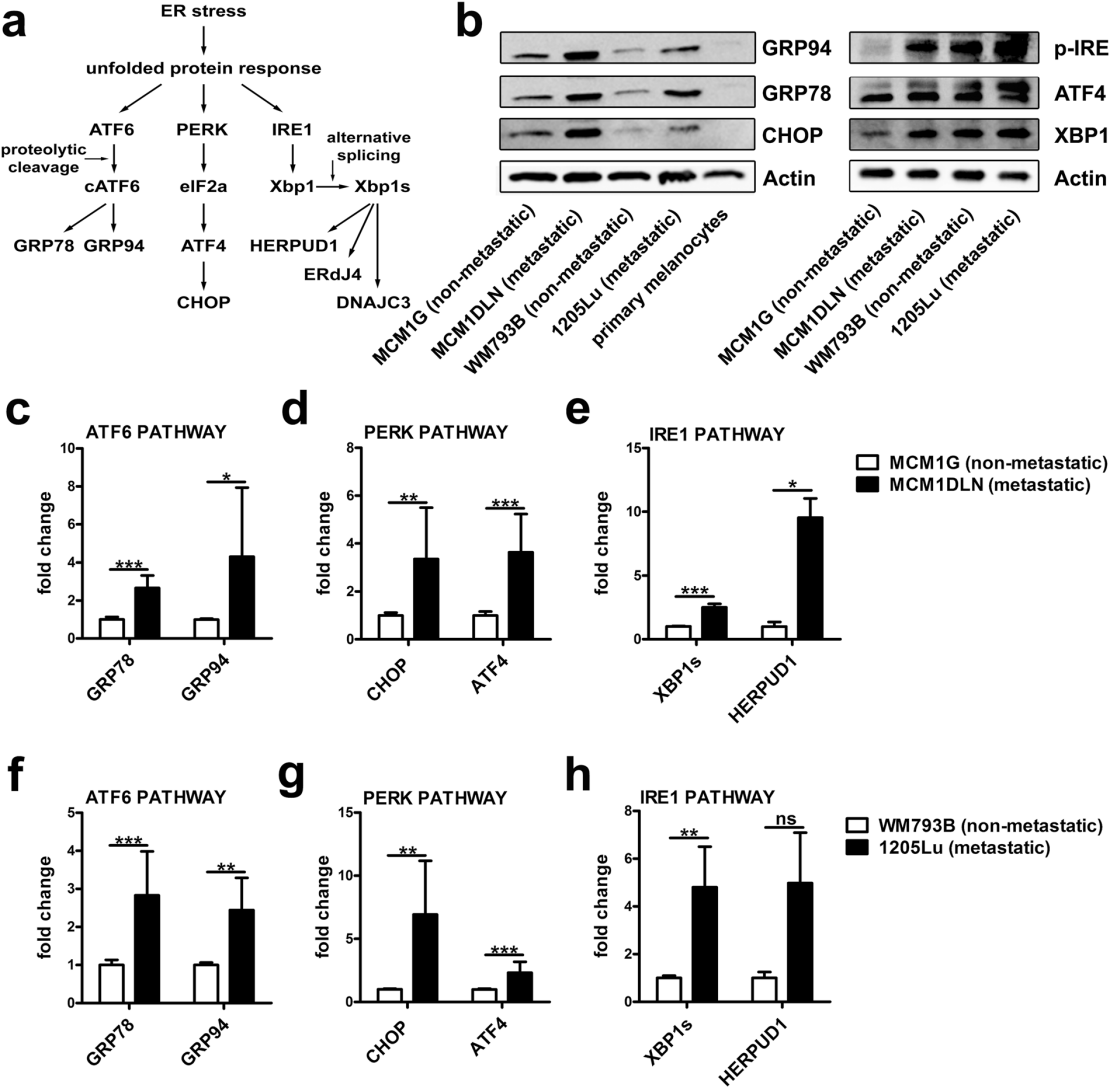
Down-stream signalling of the unfolded protein response (UPR) is enhanced in metastatic melanoma cell lines. (**a**) Illustration of the UPR and selected down-stream targets of the ATF6, PERK and IRE1 pathway. (**b**) Isogenic non-metastatic and metastatic melanoma cells and primary melanocytes were cultivated in MIM and protein expression was analyzed by immunoblotting. One representative blot out of three independent experiments is shown. Uncropped immunoblot scans are shown in supplementary Fig. S7. (**c**–**e**) mRNA expression pattern of down-stream targets of the UPR in isogenic MCM1G and MCM1DLN and (**f**–**h**) isogenic WM793B and 1205Lu cells. Cells were cultivated in MIM, expression was determined by RT-qPCR and results were normalized to β-actin. Pooled data of at least three independent experiments are shown.

The UPR is initiated by elevated endoplasmic reticulum (ER) stress and plays pivotal roles in numerous metabolic disorders such as obesity, inflammation, diabetes mellitus, the de-regulation of lipid metabolism as well as in cancer^6,7^. In various cancer types homeostasis in the ER is disturbed due to a higher protein synthesis burden, nutrient deprivation or hypoxia and consequently the UPR is induced to enable adaptive mechanisms to restore ER homeostasis. Glucose-regulated protein 78 (GRP78, also known as BiP), a master regulator of the UPR, is up-regulated in several cancers, including melanoma, breast, and prostate cancer^8^. During the UPR several translational and transcriptional programmes and the synthesis of ER chaperones are induced^9^. These adaptive mechanisms are regulated by three ER resident proteins: ATF6 (activating transcription factor 6), PERK (eukaryotic translation initiation factor 2-alpha kinase 3) and IRE1 (serine/threonine protein kinase/endoribonuclease IRE1). These proteins are localized in the ER and are bound to GRP78. Upon ER stress induction, GRP78 is released from ATF6, PERK and IRE1, which initiates down-stream signalling as illustrated in Fig. 1a. Subsequently, the UPR increases the ER folding capacity by induction of chaperones via the ATF6 branch. Further, inhibition of translation via PERK and IRE1 leads to the reduction of protein folding burden in the ER. The latter branch is dependent on alternative splicing of XBP1 to generate spliced XBP1 (XBP1s) as an active transcription factor. The overall consequence of the UPR induction is to re-establish ER homeostasis or to induce apoptosis via the PERK-target CHOP, if adaptive mechanisms fail^9^.

The decision between survival and apoptosis of a cell is dependent on whether ER stress can be mitigated and homeostasis of the ER can be re-established in time^10^. The UPR’s dual function in adaption and apoptosis poses the questions whether activation of the UPR promotes cancer progression or whether it has to be considered a tumour suppressor. On the one hand, oncogenic activation of the mitogen-activated protein kinase/extracellular signal-regulated kinase (MEK/ERK) triggers protein synthesis, resulting in an adaptive UPR in melanoma cells, which promotes proliferation and protects against apoptosis^11^. On the other hand, pharmacological induction of the UPR was suggested as an anti-tumour strategy: In BRAF^V600E^ melanoma cells, vemurafenib has anti-tumour activity by promoting ER stress-mediated apoptosis^12^. Moreover, HA15, a compound that specifically targets GRP78, induces ER stress leading to cancer cell death *in-vitro* and *in-vivo*, even in BRAF inhibitor resistant melanoma cells^13^.

In these studies, together with numerous studies using chemical inducers of the UPR like thapsigargin or tunicamycin^14,15^, the UPR is activated to levels that exceed the intrinsic conditions in tumours. Hence, these findings do not necessarily contribute to the question, whether the UPR is pro- or anti-tumorigenic *per-se*. To address this question, we studied isogenic melanoma cell lines harboring BRAF^V600E^ mutations derived from human patients as a model system to analyze intrinsic activation of the UPR. This model system allowed us to study the consequences of intrinsic UPR activity without artificially inducing ER stress and to identify FGF1 and FGF2 as novel UPR targets in melanoma contributing to its progression. Importantly, we show that melanoma ER stress can be antagonized by the chemical chaperone 4-phenylbutyric acid (4-PBA).

## Results

### The activity of the unfolded protein response (UPR) is increased in metastatic melanoma cell lines

To analyze the role of the UPR in melanoma metastasis we made use of isogenic, patient-derived human melanoma cell lines carrying the clinically relevant BRAF^V600E^ mutation. The expression of UPR-down-stream targets was analyzed in isogenic non-metastatic MCM1G and metastatic MCM1DLN cells as well as in isogenic non-metastatic WM793B and metastatic 1205Lu melanoma cells under standard culture conditions. Figure 1b shows that protein expression levels of the ER-chaperones GRP78 and GRP94 as well as expression of CHOP are increased, indicative of elevated UPR activity in metastatic MCM1DLN and 1205Lu cells compared to their non-metastatic counterparts. Expression of these proteins was considerably higher in melanoma cells compared to primary human melanocytes used as controls. Moreover, protein levels of p-IRE, XBP1 and ATF4 are up-regulated in metastatic cell lines compared to their non-metastatic counterparts (Fig. 1b). Consistently, mRNA levels of down-stream targets of all three UPR branches (ATF6, PERK and IRE1) were up-regulated in MCM1DLN compared to MCM1G cells (Fig. 1c–e). Importantly, a similar pattern of UPR activation was found in metastatic 1205Lu compared to non-metastatic WM793B cells (Fig. 1f–h). Increased UPR activity was not accompanied by increased apoptosis in metastatic melanoma cells (Supplementary Fig. S1). These data indicate an increased intrinsic UPR-activity in metastatic compared to non-metastatic melanoma cells, which does not induce apoptosis.

### Non-metastatic melanoma cells are more sensitive to the induction of acute ER stress

**Figure 2:**
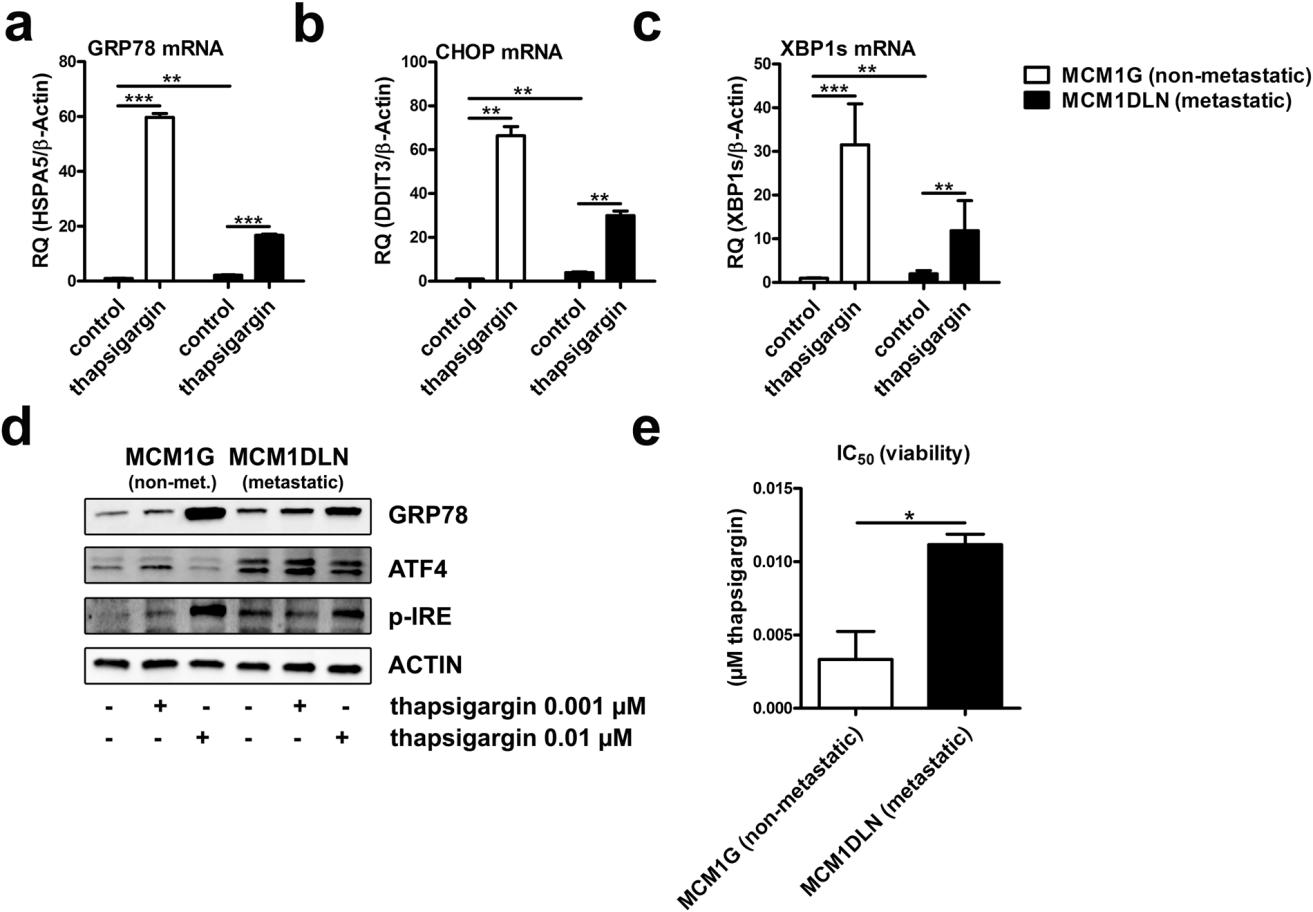
Non-Metastatic melanoma cells are more sensitive to acute ER stress induction. (**a**–**c**) Expression of down-stream targets of the UPR after acute ER stress induction (0.01 µM thapsigargin in MIM for 48 hours) was determined by RT-qPCR (n = 2). (**d**) Protein expression of each pathway of the UPR was determined by immunoblotting in isogenic non-metastatic MCM1G and MCM1DLN after thapsigargin treatment (0.001 µM and 0.01 µM in MIM for 48 hours). One representative blot out of three independent experiments is shown. Uncropped immunoblot scans are shown in supplementary Fig. S8. (**e**) Viability of isogenic non-metastatic MCM1G and metastatic MCM1DLN cells was tested using increasing concentrations of thapsigargin in MIM for 48 hours and by determining IC_50_ concentrations (n = 3).

On the one hand, increased UPR-activity might be a consequence of disturbed ER homeostasis reflecting increased vulnerability to the induction of cellular stress. On the other hand, increased UPR activity might result in enhanced metabolic flexibility to adapt to cellular stress. To discriminate between these possibilities, we induced acute disturbance of ER homeostasis using the well-characterized ER stress agent thapsigargin. Thapsigargin treatment induced additional activation of all three UPR branches with more pronounced effects in non-metastatic melanoma cells (Fig. 2a–c). On protein level, analysis of the down-stream targets of the ATF6 pathway (GRP78) and the IRE1 pathway (p-IRE) showed comparable effects upon thapsigargin treatment. Non-metastatic MCM1G melanoma cells were more sensitive to thapsigargin treatment compared to metastatic MCM1DLN cells. The down-stream target of the PERK pathway (ATF4) was down-regulated with higher thapsigargin concentration, probably because of translational inhibition due to high ER stress levels (Fig. 2d). Analysis of cell viability revealed that the IC_50_ concentration of thapsigargin was about 3 times higher in MCM1DLN cells compared to MCM1G cells (Fig. 2e). Hence, metastatic cells having higher intrinsic UPR-activity are able to compensate acute ER stress more effectively and are less sensitive to the induction of acute ER stress.

### Enhanced activity of the UPR is associated with a poor prognosis for survival in melanoma patients

**Figure 3:**
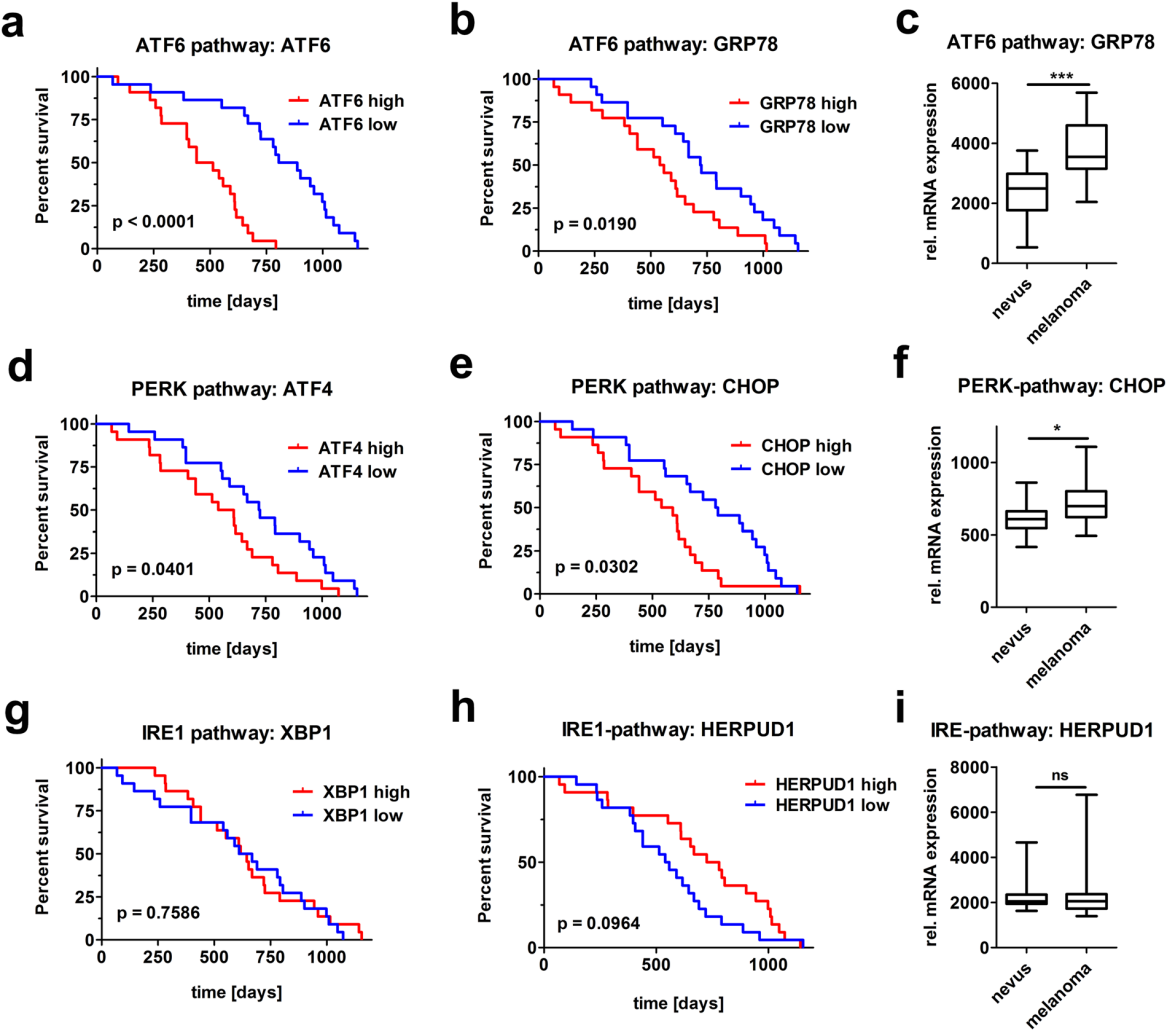
Melanoma patients with enhanced ATF6 and PERK branch activity show decreased survival. High ATF6 and PERK branch activity shows negative impact on the survival of melanoma patients. Kaplan-Meier analyses were performed on the activity of ATF6 (**a**–**b**), PERK (**d**–**e**) and IRE1 pathways (**g**–**h**) using mRNA microarray datasets previously published^16^. Expression of mRNA levels of the ATF6 (**c**), the PERK (**f**) and the IRE1 pathway (**i**) was determined in nevi and melanoma samples from datasets previously published^17^.

To evaluate the contribution of the UPR in melanoma malignancy in patients, we analyzed microarray expression data from 44 human melanoma patients^16^. Kaplan-Meier analyses show decreased survival of melanoma patients with high mRNA levels of ATF6 and its down-stream target GRP78 (Fig. 3a–b). Moreover, we found GRP78 mRNA expression to be enhanced in melanomas compared to benign nevi (Fig. 3c) by analyzing microarray data comparing melanoma and nevi in human patient samples^17^. Similarly, expression of the PERK pathway down-stream targets ATF4 and CHOP was negatively correlated with survival (Fig. 3d–e) and CHOP expression was elevated in melanoma compared to nevi samples (Fig. 3f). In contrast, high expression of the IRE1 target XBP1 was not associated with a difference of survival (Fig. 3g). Another down-stream target of the IRE1 pathway, HERPUD1, showed even better prognosis for melanoma patients when it was highly-expressed (Fig. 3e). No association of the expression of ERDJ4 or DNAJC3, two distinct IRE1-targets, with survival was observed (Supplementary Fig. S2a). In addition, none of the abovementioned IRE1 targets were increased in melanomas compared to nevi, with the exception of DNAJC3 (Fig. 3i and Supplementary Fig. S2b). Our data indicate increased activation of all three ER stress branches in metastatic melanoma cell lines. However, only enhanced activities of ATF6 and PERK, but not of the IRE1-branch, are associated with poor survival in patients.

### A chemical chaperone antagonizes UPR-activity

**Figure 4:**
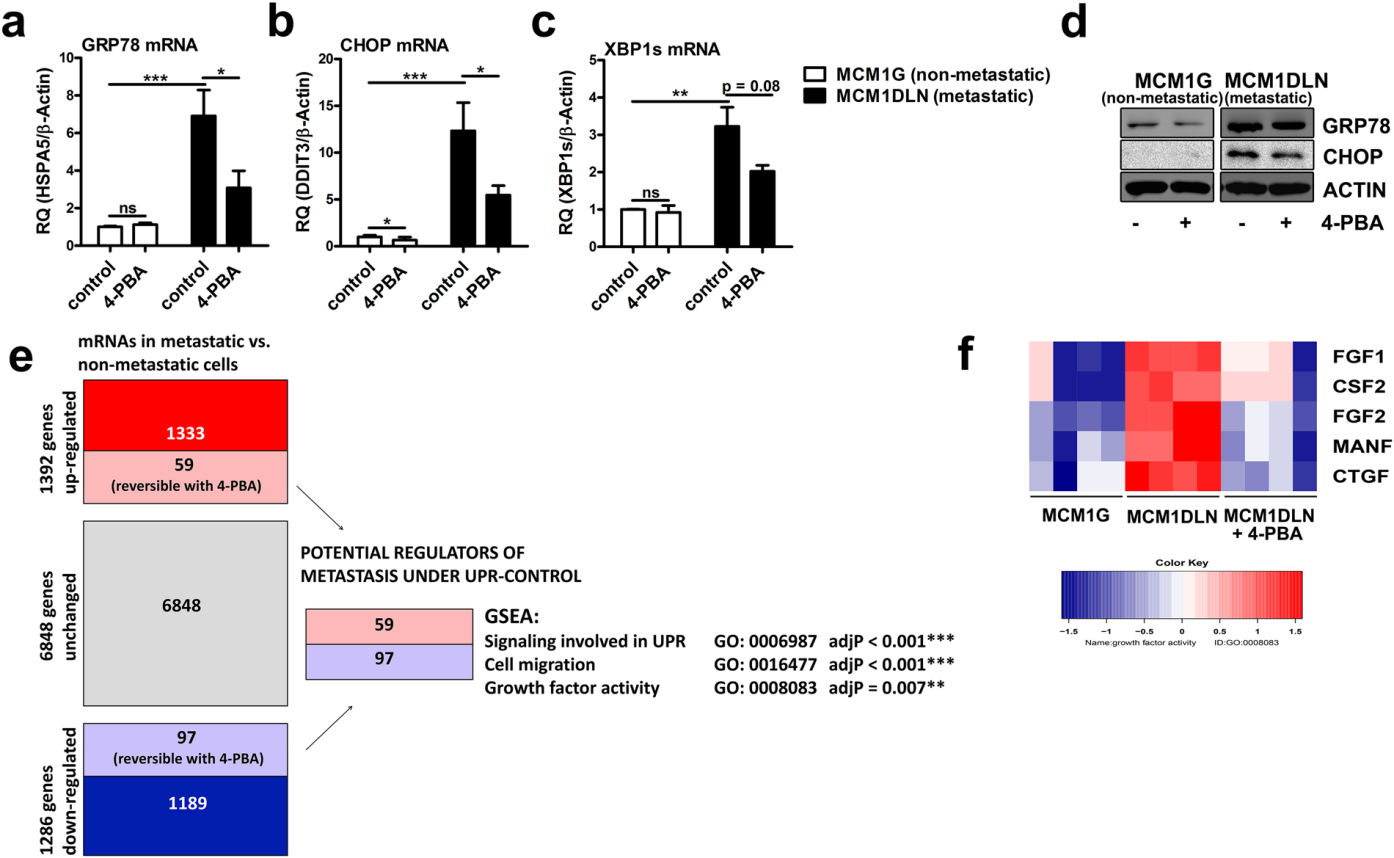
Microarray analysis and gene set enrichment analysis after antagonizing the UPR using the chemical chaperone 4-phenylbutyric acid (4-PBA) in metastatic melanoma cells. (**a**–**c**) Expression of UPR markers after ER stress reduction using 4-PBA (1 mM in MIM for 48 hours) was determined by RT-qPCR (n = 3; data shown as mean ± sem). (**d**) Protein expression of GRP78 and CHOP was determined by immunoblotting in isogenic non-metastatic MCM1G and metastatic MCM1DLN cells after 4-PBA treatment (1 mM in MIM for 48 hours). One representative blot out of three independent experiments is shown. Uncropped immunoblot scans are shown in supplementary Fig. S9. (**e**) Illustration of the microarray setup and Gene Set Enrichment Analysis (GSEA) using MCM1G, MCM1DLN, and MCM1DLN cells treated with 1 mM 4-PBA for 48 hours. (**f**) Heatmap of the mRNA expression pattern of altered growth factors in MCM1G, MCM1DLN and MCM1DLN cells treated with 1 mM PBA in MIM for 48 hours (data show two independent experiments performed in duplicates). CSF2, colony stimulating factor 2; MANF, mesencephalic astrocyte derived neurotrophic factor; CTGF, connective tissue growth factor.

The chemical chaperone 4-phenylbutyric acid (4-PBA) was previously shown to ameliorate ER stress and to reduce UPR activity especially in the liver^18^. Of note, this drug is already in clinical use to treat rare uremic cycle disorders. We therefore tested if 4-PBA is able to mitigate UPR activity in metastatic melanoma cells. Indeed, treatment of metastatic MCM1DLN cells partially reverted the enhanced activity of all three UPR branches as monitored by a decrease of GRP78, CHOP and XBP1s mRNA expression (Fig. 4a–c). Consistently, protein expression of GRP78 and CHOP was reduced by 30% and 40% (quantification of three independent experiments), respectively, in metastatic MCM1DLN cells after 4-PBA treatment (Fig. 4d).

To analyze the functional consequences of UPR-activation in metastatic melanoma cells, we designed an experimental setting to identify putative metastasis-modulating genes regulated by the UPR. Thus, we performed RNA microarray analysis of genes differentially regulated between MCM1G and MCM1DLN cells. In addition we analyzed, which of these differential regulations could be reverted by 4-PBA treatment (Fig. 4e). We identified 59 genes up-regulated in metastatic cells that were down-regulated by 4-PBA, representing potential drivers of melanoma metastasis under control of the UPR (Supplementary Dataset 1). Moreover, we identified 97 genes down-regulated in metastatic cells that were in turn up-regulated by 4-PBA treatment, representing putative inhibitors of metastasis under control of the UPR (Supplementary Dataset 2). After gene set enrichment analysis (GSEA) on these 156 differentially regulated genes, “activation of signalling involved in the UPR” was the pathway enriched most significantly. This expected finding is indicative of the validity of the experimental approach. Intriguingly, genes involved in “cell migration” and “growth factor activity” were significantly enriched after adjustment for multiple testing (Fig. 4e). In fact, several growth factors were up-regulated in metastatic MCM1DLN cells and reduced after 4-PBA treatment (Fig. 4f), including FGF1 and FGF2. The relative expression of all FGFs and FGF-receptors are shown in supplementary Figure S3. Given the pivotal role of FGFs in mediating cancer progression, we selected these growth factors and their regulation by the UPR for further analysis.

### 4-PBA reduces FGF1 and FGF2 expression and invasion in metastatic melanoma cells

**Figure 5:**
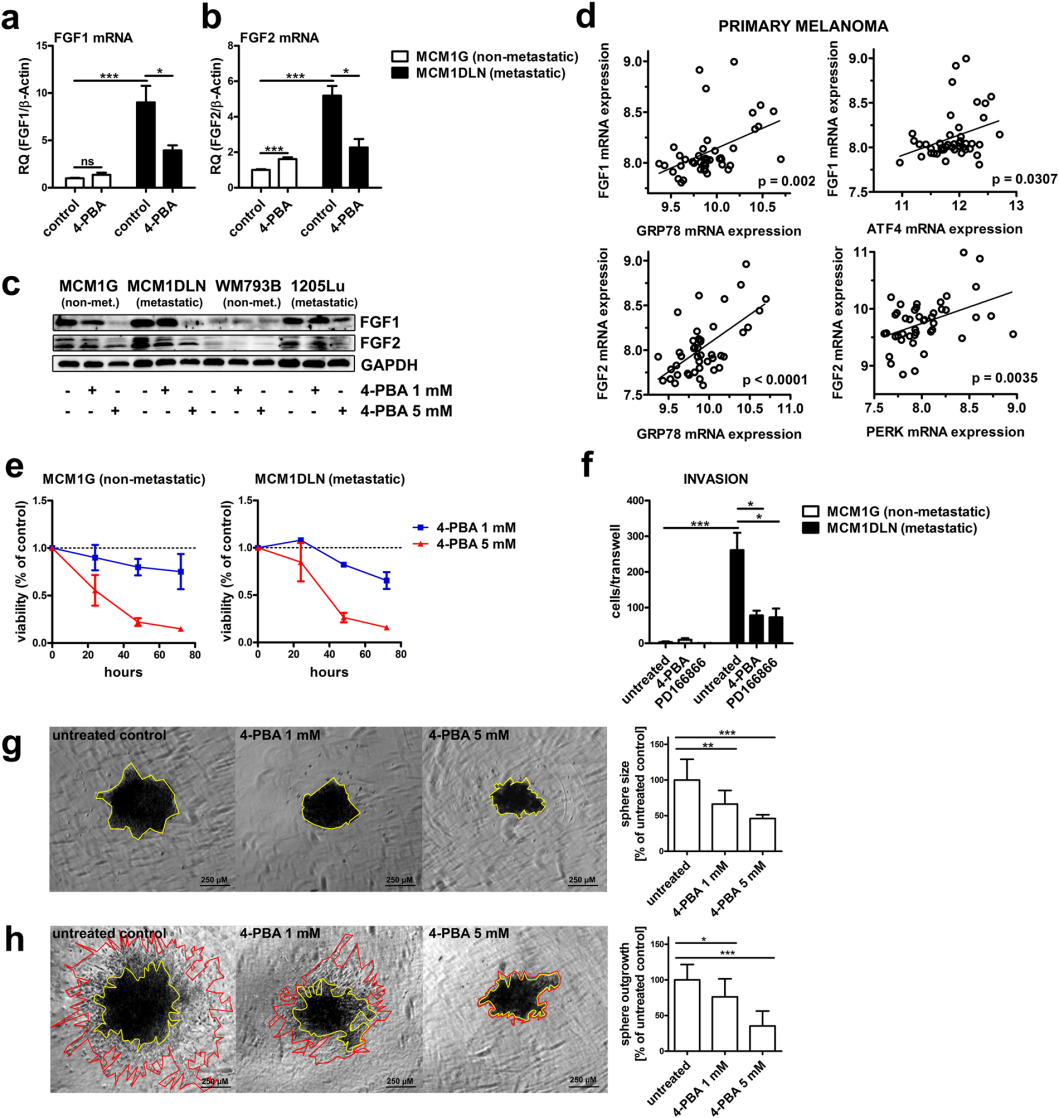
FGF1 and FGF2 expression and invasion is diminished by 4-PBA in metastatic melanoma cells. (**a**–**b**) Expression of FGF1 and FGF2 after UPR-reduction using 1 mM 4-PBA for 48 hours was determined by RT-qPCR (data shown in mean ± sem). (**c**) Protein expression of FGF1 and FGF2 after ER stress reduction using 4-PBA for 48 hours was determined in isogenic non-metastatic MCM1G and metastatic MCM1DLN cells and in isogenic non-metastatic WM793B and metastatic 1205Lu cells by immunoblotting. One representative blot out of three independent experiments is shown. Uncropped immunoblot scans are shown in supplementary Fig. S10. (**d**) Correlation of the mRNA expression of down-stream targets of the UPR with FGF1 and FGF2 was determined using microarray datasets from a previously published study^16^. (**e**) Viability assay of 1 mM and 5 mM 4-PBA treated MCM1G and MCM1DLN cells in MIM for the indicated time points (n = 3; data shown in mean ± sem). (**f**) Invasion assay of isogenic MCM1G and MCM1DLN cells pre-treated with 1 mM 4-PBA and 10 µM PD166866 for 16 hours in MIM, followed by 8 hours invasion through matrigel-coated transwells in MIM without FBS and 0.1% BSA containing the described treatments (n = 3; data shown in mean ± sem). (**g**) MCM1DLN sphere size areas with or without treatment with 4-PBA were evaluated after 72 hours in MIM containing 20% methylcellulose (n = 3; data shown in mean ± SD). For each treatment condition one representative image is shown. Yellow: spheroid. (**h**) Sphere outgrowth distance was determined after 24 hours in collagen gels treated with or without 4-PBA in MIM. Spheroids were pre-treated with 4-PBA during sphere formation for 72 hours MIM containing 20% methylcellulose (n = 3; data shown in mean ± SD). For each treatment condition one representative image of sphere outgrowths into collagen after 24 hours is shown. Yellow: spheroid; red: border of spheroid outgrowths.

Microarray and GSEA data were validated by examining FGF1 and FGF2 expression in non-metastatic and metastatic melanoma cells after 4-PBA treatment. FGF1 and FGF2 mRNA levels were significantly increased in metastatic MCM1DLN cells compared to non-metastatic MCM1G cells and decreased in metastatic MCM1DLN cells after 4-PBA treatment (Fig. 5a–b). Analysis of protein levels showed increased levels of FGF1 and FGF2 in isogenic metastatic MCM1DLN cells compared to non-metastatic MCM1G cells and displayed a dose-dependent reduction of FGF1 and FGF2 upon 4-PBA treatment in both pairs of cell lines. Importantly, FGF1 and FGF2 levels were also increased in metastatic 1205Lu compared to non-metastatic WM793B cells and were likewise decreased by 4-PBA treatment (Fig. 5c). In primary tumours of human melanoma patients, the expression of both FGF1 and FGF2 mRNA displayed a significant positive correlation with the expression of down-stream targets of the ATF6 and PERK branches of the UPR (Fig. 5d). This points towards a general regulatory mechanism of FGF1 and FGF2 expression by the UPR. No correlation of FGF1 and FGF2 expression with down-stream targets of the IRE1-pathway was observed (Supplementary Fig. S4).

As our GSEA revealed a potential functional role of the UPR in regulating growth factor activity and cell migration, we performed functional validations. Analysis of cell viability revealed a dose-dependent reduction after 4-PBA treatment in both metastatic and non-metastatic cells (Fig. 5e). Notably, the specific FGF1-receptor inhibitor PD166866 likewise reduced cell viability indicating dependency on the FGF-receptor pathway (Supplementary Fig. S5). Furthermore, we analyzed cells’ invasive potential by assessing invasion and migration through matrigel-coated transwells. Invasion was barely detected for non-metastatic MCM1G cells. In contrast, invasion was high in metastatic MCM1DLN cells and was reverted by 4-PBA treatment (Fig. 5f). Of note, these effects were observed after treatment with 1 mM PBA for 24 hours, which did not affect cell viability (compare Fig. 5e). Importantly, the FGF1 receptor inhibitor PD166866 exerted similar effects on cell invasion (Fig. 5f) and therefore FGF/FGF-R signalling is essential for invasion of melanoma cells. Additionally, we analyzed the effects of 4-PBA on sphere size and sphere outgrowth in a 3D cell-model by generating MCM1DLN spheroids. We observed a dose-dependent decrease in size of 4-PBA treated spheres (Fig. 5g) as well as a dose-dependent reduction in sphere outgrowths into collagen (Fig. 5h). Taken together, the UPR regulates FGF1 and FGF2 expression in human melanoma and reduction of the UPR using a chemical chaperone reduces cell viability and invasion.

Finally we aimed to translate our *in-vitro* findings to a pre-clinical setting. Therefore, we orally applied 4-PBA to mice xenografted with MCM1DLN or 1205Lu cells. However, 4-PBA failed to reduce UPR activity in tumours and therefore no beneficial effects on tumour progression were observed (Supplementary Fig. S6).

## Discussion

We utilized isogenic, patient-derived melanoma cells to study the role of the UPR in melanoma progression. Specifically, we described the UPR to be up-regulated in metastatic compared to non-metastatic cell lines. Further, we characterized the pathophysiological consequences of UPR elevation using the chemical chaperone 4-PBA to antagonize the UPR. The identified cell models enable the characterization of intrinsic UPR-activity without the necessity to induce artificial acute ER stress by chemical inducers such as thapsigargin or tunicamycin. Although these compounds are valuable experimental tools, their potential to induce ER stress exceeds physiological conditions and may thus lead to artificial findings. Excess, acute ER stress ultimately leads to apoptosis, whereas chronic ER stress is more modest and demands adaptive mechanisms to persistently tolerate prolonged UPR signalling. Thus, chronic ER stress has to be regarded as a pathway mediating adaption to stressful stimuli^19^.

In cancer, ER stress and the UPR can be triggered by a variety of factors including inadequate vascularization, rapid tumour growth, hypoxia, nutrient deprivation, reduced glycosylation of secretory proteins and increased protein folding burden^5,20^. In addition, aberrant regulation of growth factor-mediated signalling pathways induce the UPR. Activation of mTORC1, by loss of its inhibitor TSC2, results in elevated UPR^21^. In melanoma cells, the commonly hyper-activated signalling pathways MEK/ERK as well as RAS and RAF mediate increased protein synthesis via mTORC1 leading to enhanced UPR^11,22^.

The consequence of UPR-activation is either metabolic adaption or apoptosis, if homeostasis of the ER cannot be restored. This process is delicately balanced and the biological consequence seems to depend on the level of UPR activity: The action of PERK in a BRAF-activated background can function either as a tumour suppressor or tumour promoter, depending on gene dosage^23^. Heterozygous deletion of the PERK gene is permissive for BRAF^V600E^-dependent transformation, while complete deletion of both PERK alleles is tumour suppressive^23^. In untransformed cells under high levels of acute ER stress, the PERK pathway increases protein synthesis and thus mediates cell death. Hence, the activity of the UPR, in particular the activity of PERK, needs to be finely tuned to ensure protein folding homeostasis in the ER^24^. In addition to that, the timing of UPR activation in the course of tumour formation is relevant. Oncogene-driven induction of the UPR is anti-oncogenic in early phases of melanocyte transformation^25^. In already transformed melanoma cells, MEK/ERK-driven activation of the UPR promotes proliferation^11^. Moreover, BRAF-inhibitor-induced ER stress activates cytoprotective apoptosis^26^. Our data suggest that the level of UPR discriminates metastatic from non-metastatic cells. In addition, we found that high activity of the UPR is associated with poor prognosis in melanoma patients. Altogether, this indicates that increased UPR signalling is an adaptive mechanism driving the aggressiveness of advanced melanoma, but not necessarily of pre-tumorigenic nevi. Our melanoma cell line pairs therefore represent a good model for the investigation of chronic, intrinsic ER stress, which is apparent in melanoma metastasis.

Under physiological conditions, prolonged UPR activity triggers apoptosis. Apparently, melanoma cells have found ways to circumvent UPR-mediated apoptosis. Indeed, BRAF^V600E^ mutated melanoma cells adapt to chronic ER stress conditions via increased basal autophagy to circumvent apoptosis^27^. In normal cells, the activity of IRE1 is diminished under persistent ER stress leading to PERK-mediated apoptosis^28^. In melanoma cells, however, IRE1 activity is not attenuated by prolonged, pharmacological ER stress, but sustained via the MEK/ERK pathway and can therefore counteract PERK-mediated apoptosis^29^. In addition to cell type specific regulation, the induction of IRE1 activity may depend on the environmental background: Expression of BRAF^V600E^ in primary melanocytes induced IRE1-activity *in-vitro*, however, its activity is diminished in the skin of BRAF^V600E^-transgenic mice^23^. This might also explain our observed activation of all three UPR branches in metastatic compared to non-metastatic cells, while only the induction of the ATF6- and PERK-, but not the IRE1-pathway, is associated with poor survival in melanoma patients.

Hyper-activation of the FGF/FGFR-signalling axis is closely associated with the progression of many cancer types. Different human melanoma cell lines constitutively express a diverse pattern of growth factors, but share common expression of FGF2^30^. FGF-overexpressing melanocytes exhibit pro-tumorigenic properties like increased proliferation and migration^31^. Consistently, disruption of the FGFR/FGF-signalling axis displayed antitumor-activity of melanoma *in-vivo* and *in-vitro* ^32^. Our data show that the UPR in metastatic melanoma cells contributes to increased FGF1 and FGF2 expression and enhances cell migration. Mechanistically, it was described that FGF2 drives melanoma cell migration through a syndecan-4 and focal adhesion kinase-dependent mechanism^33^. This finding together with the fact that we could diminish invasion and migration by FGFR1-inhibition strongly suggests that the UPR contributes to a migratory phenotype predominantly via an FGF2/FGFR1-dependent mechanism. Consistent with our *in-vitro* data we found a significant positive correlation of UPR-activity and FGF expression in primary human melanomas. Interestingly, activation of PERK induced FGF2 expression in independent model systems as shown in hypoxic muscle and in cancer cells following glucose deprivation^34,35^, pointing towards a general regulatory mechanism.

Melanoma metastasis requires an EMT-like process referred to as “phenotype switching” which is characterized by reduction of the proliferative potential, while the migratory and invasive potential rises^36^. Several lines of evidence link ER stress to a migratory and invasive phenotype: GRP78 expression is especially high at the invasive front of human melanomas^37^. Consequently, a monoclonal antibody against GRP78 suppresses PI3K/AKT signalling, tumour growth and metastasis in cancer cells^38^. In line, PERK is activated upon EMT activation in breast-cancer cells^39^. We thus hypothesize that our observed up-regulation of FGFs by the UPR is an essential step in phenotype switching that enhances melanoma aggressiveness by mediating a migratory and invasive phenotype.

4-PBA has an excellent *in-vivo* safety profile, is approved by the FDA for clinical use in rare uremic cycle disorders^40^ and exerts beneficial metabolic effects in the liver in pre-clinical studies^18^. In addition, it was shown to inhibit tumour growth in pancreatic and prostate cancer^41,42^. However, this drug is highly water-soluble and characterized by an unfavorable pharmacokinetic and pharmacodynamic profile^43^. In our isogenic-metastatic melanoma cell models we found a reduction of down-stream targets of the UPR after 4-PBA treatment. Furthermore, using this chaperone, we revealed a link between the UPR and fibroblast growth factors, as FGF1 and FGF2 are up-regulated in metastatic melanoma cells and can be reduced after 4-PBA treatment. Further, 4-PBA reduced cell viability and migration in a 2D and 3D setting. However, relatively high doses of 4-PBA in the micro-molar range were necessary to observe these effects indicating a low potency of this chaperone. In addition, 4-PBA failed to reduce the UPR in xenograft models of melanoma (Supplementary Fig. S6). Insufficient drug delivery to the tumour, drug inactivation by metabolization or low potency might limit the readout of this *in-vivo* experiment. Given the low potency and the unfavorable pharmacokinetic and pharmcodynamic profile, the synthesis of more potent 4-PBA analogues have been described^44^, however they have not been sufficiently characterized *in-vitro* and *in-vivo* yet. More potent and specific inhibitors for individual UPR branches, particularly for the PERK pathway are available, but have severe side effects against secretory tissues, especially against the pancreas^45^.

The dual role of ER stress ultimately poses the question whether induction or the reduction of the UPR response is a promising approach to treat human melanoma. Several studies have shown that the induction of ER stress is a promising approach towards treatment of melanoma in xenograft models^12,13^. In contrast, our data show that metastatic cells with increased UPR activity tolerate increased concentrations of the ER stress-inducing agent thapsigargin. In line with our finding, the induction of the UPR represents an adaptive mechanism to ER stress^11^.

Moreover, IRE1-mediated activation of AKT confers resistance against docetaxel and vincristine in melanoma cells after induction of ER stress^46^. This suggests that malignant melanoma cells have adapted themselves to cope with ER stress and in contrast benign cells are more susceptible to cell death upon ER stress induction. We hypothesize that in heterogeneous human melanoma the pharmaceutical induction of ER stress might preferentially target benign tumour cells and thus confers a selection advantage for malignant cells. Therefore we hypothesize that the reduction of ER stress using chemical chaperones is a more promising strategy to target malignant cells in order to reduce melanoma metastasis. This will require the identification of more potent chemical chaperones with favorable pharmacokinetic properties, which are not available yet. As the activation of the UPR is not limited to melanoma, but rather a broad phenomenon in various cancer types, we anticipate that the general use of potent chemical chaperones, together with tumour type specific targeted therapy approaches, is a promising strategy to fight cancer.

## Materials and Methods

### Ethical Considerations

All experiments and analyses were conducted in accordance with Austrian laws and guidelines. Animal experiments were approved by the Austrian Ministry of Science, Research and Economy (licence#: BMWFW-66.009/0117-WF/V/3b/2015). Human cell lines and primary melanocytes were either obtained commercially (WM793B, 1205Lu and primary melanocytes) or isolated from a human patient as described below (MCM1G and MCM1DLN). The procedure was approved by the Ethics Committee of the Medical University of Vienna (licence#: EK 093/2003 and 191/05/2009). Informed consent was obtained from all subjects.

### Melanoma Cell lines

Isogenic non-metastatic MCM1G and metastatic MCM1DLN melanoma cells were isolated from the same patient and characterized *in-vivo* as previously described^47^. Isogenic non-metastatic WM793B and metastatic 1205Lu melanoma cell lines, which are derived from another single patient, were obtained from the American type Culture Collection (ATCC, Manassas, USA). Primary human melanocytes were obtained from PromoCell (PromoCell, Heidelberg, Germany).

Cells identity was regularly verified and the absence of mycoplasma infections was regularly confirmed using the LONZA MycoAlert PLUS mycoplasma detection kit (Lonza Group Ltd, Basel, Switzerland).

### Cell culture

Cells were cultivated under standard conditions (37 °C, 5% CO_2_) and grown in melanoma isolation media (MIM): MCDB153 medium / Leibovitz’s L-15 medium (4/1) supplemented with 2% FBS, 7.5% NaHCO_3_, 5 µg/ml Insulin, 5 ng/ml EGF, 1.68 mM CaCl_2_, 50 mg/L streptomycin sulphate and 30 mg/L penicillin. Cells were split when 90% confluent using a 0.25% trypsin/EDTA solution. If not otherwise indicated, experiments were performed in MIM for 48 hours. 4-phenylbutyric acid (4-PBA) was obtained from Santa Cruz Biotechnology (Dallas, USA) and PD166866 and thapsigargin were obtained from Sigma-Aldrich (St. Louis, USA).

### Gene expression analysis

RNA was isolated using the peqGOLD Total RNA kit (Peqlab, Erlangen, Germany) and cDNA was synthesized from 0.5 µg RNA using the high capacity cDNA reverse transcription kit (Thermo Fisher Scientific, Waltham, USA). For RT-qPCR the iTaq^TM^ universal probes supermix (BIO-RAD, Hercules, USA) was used according to the manufacturer’s protocol. Primers were purchased from Thermo Fisher Scientific (Supplementary Table S1). Data were evaluated using StepOne Software v2.3 (Thermo Fisher Scientific).

### Western Blot

Immunoblotting was performed according to standard methods. Cells were lysed in Ripa lysis buffer (Merck, Darmstadt, Germany), supplemented with 1% protease inhibitor cocktail (Sigma-Aldrich, St. Louis, USA) and 1% phenylmethylsulfonyl fluoride (Sigma-Aldrich). Protein concentration was determined by Bradford protein analysis (Bradford reagent, BIO-RAD). Proteins were separated on a 12% (w/v) SDS-PAGE and transferred onto a 0.45 µM nitrocellulose membrane (BIO-RAD). The primary antibodies used for the experiments are summarized in supplementary Table S2. The appropriate horseradish peroxidase-coupled secondary antibodies were incubated in 5% (w/v) milk/BSA in TBS-T (Goat Anti-Mouse IgG (H + L)- HRP Conjugate (BIO-RAD); Goat Anti-Rabbit IgG (H + L)- HRP Conjugate (BIO-RAD)) for 1 hour at room temperature, followed by detection of the proteins using the SuperSignal West Dura Extended Duration Substrate (Thermo Scientific) and a ChemiDoc^TM^ Touch Imaging System (BIO-RAD). Uncropped blots are shown in supplementary Figs S7–S10.

### Cell ability assay

50.000 cells per well were seeded in a 96 well plate (Greiner Bio-One, Kremsmünster, Austria) and maintained in MIM. The next day cells were treated with 4-phenylbutyric acid for the indicated time points or increasing concentrations of thapsigargin for 48 hours. Afterwards cells were incubated with MTT (3-(4,5-dimethyldiazol 2-yl)-2,5 diphenyltetrazolium bromide solution; Sigma-Aldrich) diluted in serum-free MIM for 1 hour under standard conditions (37 °C, 5% CO_2_). Cells were then washed and incubated with dimethyl sulfoxide (Sigma-Aldrich) for 10 minutes. Absorbance was measured at 490 nm and at 655 nm for reference (iMARK^TM^ microplate reader, BIO-RAD). IC_50_ values were determined from thapsigargin dose curves ranging from 6.25 nM to 0.8 µM after non-linear regression using the GraphPad Prism software (GraphPad Software, San Diego, USA).

### Microarrays and Bioinformatics

For microarray analysis, 100 ng of total RNA were hybridized to PrimeView Human Gene Expression Arrays (Affymetrix, ATLAS Biolabs GmbH, Berlin, Germany). Raw data were processed with a well-established pipeline including quantile-normalization, summarization with FARMS and improved probe set annotations^48^. Noisy genes were removed based upon their informative/non-informative (I/NI) calls as described^48^. Thereby, non-informative expression data of 8978 genes were removed. Raw data and processed data in conformation with the MIAME-criteria have been deposited in NCBI’s Gene Expression Omnibus and are accessible through GEO Series accession number GSE98023 (https://www.ncbi.nlm.nih.gov/geo/query/acc.cgi?acc=GSE98023). Gene set enrichment analysis was performed using WebGestalt^49^.

Microarray expression data from primary tumours of 44 human melanoma patients^16^ were retrieved from NCBI GEO (accession #GSE19234) and raw data were processed as described above. Microarray data from human melanoma and nevi tissues^17^ were retrieved from NCBI GEO (accession #GSE3189) as already processed data.

### Invasion Assay

Cells were pre-treated with 4-PBA and PD166866 (Sigma-Aldrich) and maintained in MIM for 16 hours. Afterwards cells were detached and re-suspended in MIM without FBS and 0.1% BSA containing the described treatments. 25.000 cells were then seeded onto matrigel invasion chambers (Corning, New York, USA), which were prepared according to the manufacturers protocol. After 8 hours, cells were fixed with formaldehyde (Sigma-Aldrich), stained with crystal violet (Sigma-Aldrich) and invading cells were counted under the microscope.

### Spheroid formation assay

5.000 cells per well were seeded in a round-bottom 96-well plate (SPL Life Sciences Co., Gyeonggi-do, Korea) and maintained in MIM containing 20% methylcellulose (Sigma-Aldrich) with or without treatment of 4-PBA. After 72 hours, spheroids were embedded into gels containing 2.5 mg/ml collagen (Thermo Scientific) and 0.1 M Hepes (Sigma-Aldrich) in PBS. Gels polymerized at standard conditions (37 °C, 5% CO_2_) for 1 hour. Afterwards gels were treated again with or without 4-PBA in MIM for 24 hours. Spheres were analyzed using the EVOS Cell Imaging System Microscopy (Thermo Scientific). Sphere size area was quantified using ImageJ 1.47 v (NIH, Bethesda, MA, USA). Sphere outgrowth distance was calculated from area data obtained from ImageJ and normalized to spheroid size after 72 hours of spheroid formation in MIM containing 20% methylcellulose.

### Statistical analysis

If not otherwise indicated, data derived from at least 3 independent experiments are depicted as the mean ± standard deviation (SD). Statistical analyses were carried out using GraphPad Prism Software. 2-sided t-tests or ANOVA followed by Tukey’s multiple testing were used to compare two or more groups, respectively. Log-rank (Mantel-Cox) test was used to analyze Kaplan-Meier plots. Correlations were analyzed using Pearson’s correlation. Significant p-values are indicated as *(<0.05), **(<0.01) or ***(<0.001).

### Data availability

The datasets generated and analyzed during the current study are available from the corresponding author on reasonable request.

## Electronic supplementary material

Supplementary material

Dataset 1

Dataset 2
