# Supplementary material for: The unfolded protein response impacts melanoma progression by enhancing FGF expression and can be antagonized by a chemical chaperone
Source: Sci Rep. 2017 Dec 13;7:17498. doi: 10.1038/s41598-017-17888-9 (PMC5727496; doi:10.1038/s41598-017-17888-9)
Supplement: Supplementary file 1 — Supplementary material [file 41598_2017_17888_MOESM1_ESM.pdf]

Supplementary material

The unfolded protein response impacts melanoma progression by enhancing FGF expression and can be antagonized by a chemical chaperone.

Short title: **UPR and FGFs in melanoma**

Karin Eigner[1], Yüksel Filik[1], Florian Mark[1], Birgit Schütz[1], Günter Klambauer[2], Richard Moriggl[3,4,5], Markus Hengstschläger[1], Herbert Stangl[1], Mario Mikula[1] and Clemens Röhrl[1]*

[1]Center for Pathobiochemistry and Genetics, Medical University of Vienna, Austria;
[2]Institute of Bioinformatics, Johannes Kepler University Linz, Austria;
[3]Ludwig Boltzmann Institute for Cancer Research, Vienna, Austria;
[4]Institute of Animal Breeding and Genetics, University of Veterinary Medicine, Vienna, Austria;
[5]Medical University of Vienna, Austria.

Supplementary methods

Flow cytometry

Cell cycle distribution of non metastatic MCM1G and metastatic MCM1DLN cells was assessed by flow cytometry after incubating cells in MIM for 48 hours. Cells were fixed in 85% ethanol at -20°C for 24 hours and afterwards stained using propidium iodide (Sigma-Aldrich, St. Louis, USA) for 20 minutes. After the incubation time cells were re-suspended in PBS, measured on the FACS CytoFlexS (Beckman Coulter Inc., Brea, USA) and results were analyzed using the CytExpert 1.0 software.

Xenograft experiment

A total of 2 x 10^6 MCM1DLN or 1205Lu melanoma cells were intradermally injected into the upper flanks of 7 weeks old CB.17 SCID mice (n=12 for each cell line). After one week, 6 mice per xenografted cell line orally received 4-PBA via drinking water resulting in a daily intake of 1g/kg body weight. Tumour volume was measured throughout the experiment using a caliper and calculated using the equation: (width x width x length)/2. Tumours were excised when they reached a size of 800 mm^3. Afterwards, 4-PBA treatment was continued until endpoint criteria were reached. Mice were sacrificed and examined for occurrence of metastases. The experiment was conducted in accordance with Austrian laws and guidelines. Experiments were approved by the Austrian Ministry of Science, Research and Economy (licence#: BMWFW-66.009/0117-WF/V/3b/2015).

Supplementary results

Supplementary Table S1. Taqman primer for RT-qPCR. Assays were purchased from Thermo Fisher Scientific (Waltham, MA, USA).

**primer**	**official gene name**	**taqman ID**
β-actin	ACTB	Hs99999903_m1
CHOP	DDIT3	Hs01090850_m1
ATF4	ATF4	Hs00909569_g1
GRP-78	HSPA5	Hs00607129_m1
FGF1	FGF1	Hs01092738_m1
FGF2	FGF2	Hs00266645_m1
ATF6	ATF6	Hs00232586_m1
XBP1s	XBP1s	Hs03929085_g1
GRP94	HSP90	Hs00427665_g1
HERPUD1	HERPUD1	Hs01124269_m1

Supplementary Table S2. Antibodies for immunoblotting

antibody	company	product #	dilution
β-Actin	Abcam	ab8229	1: 10000
GRP78	Santa Cruz Biotechnology	sc-376768	1:1000
GRP94	Santa Cruz Biotechnology	sc-393402	1:1000
CHOP	Santa Cruz Biotechnology	sc-793	1:1000
FGF1	Santa Cruz Biotechnology	sc-7910	1:500
FGF2	Merck	#05118	1:500
ATF4	Abcam	ab1371	1:1000
p-IRE1	Abcam	ab48187	1:1000
XBP1	Abcam	ab37152	1:1000

Supplementary Figure S1.

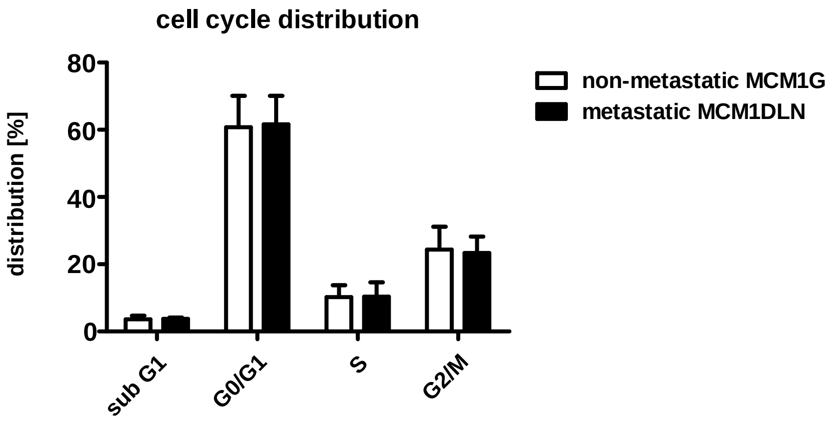

Supplementary Figure S1. Cell cycle distribution of melanoma cells. In FACS analysis, no significant differences of cell cycle distribution was observed between non-metastatic MCM1G and metastatic MCM1DLN melanoma cell lines. Sub G1 phase, also referred to as apoptotic phase, was low in both melanoma cell lines and no significant differences between non-metastatic and metastatic cells were observed (n=3).

Supplementary Figure S2.

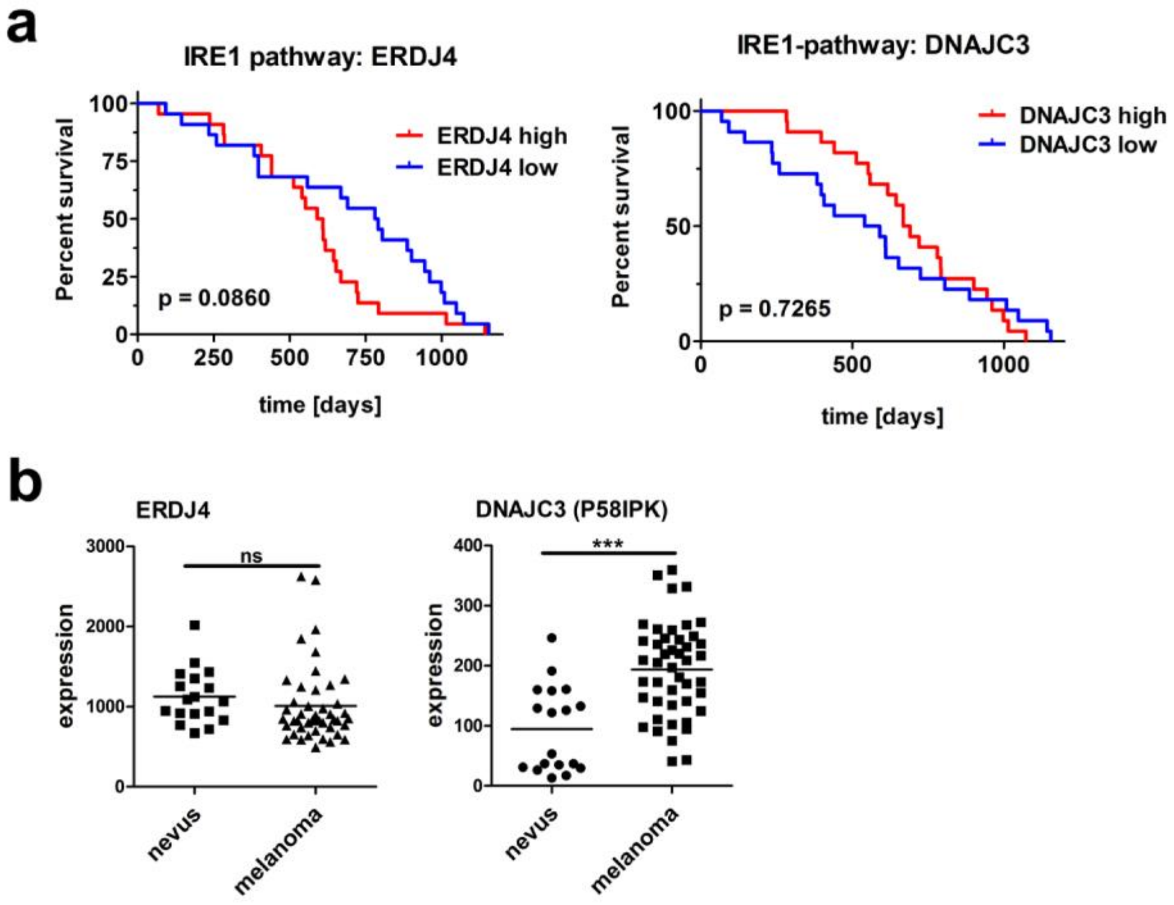

Supplementary Figure S2. Association of IRE1-activity with survival. (Fig. S2a) Kaplan-Meier analyses showed no association of ERDJ4 or DNAJ3 with survival of melanoma patients. (Fig. S2b) No significant difference in mRNA expression of ERDJ4 was observed in nevi compared to melanoma patient samples. Another IRE1 target, DNAJC3 was significantly up-regulated in melanoma samples. Data were retrieved from gene expression arrays of human patient samples[1,2].

Supplementary Figure S3.

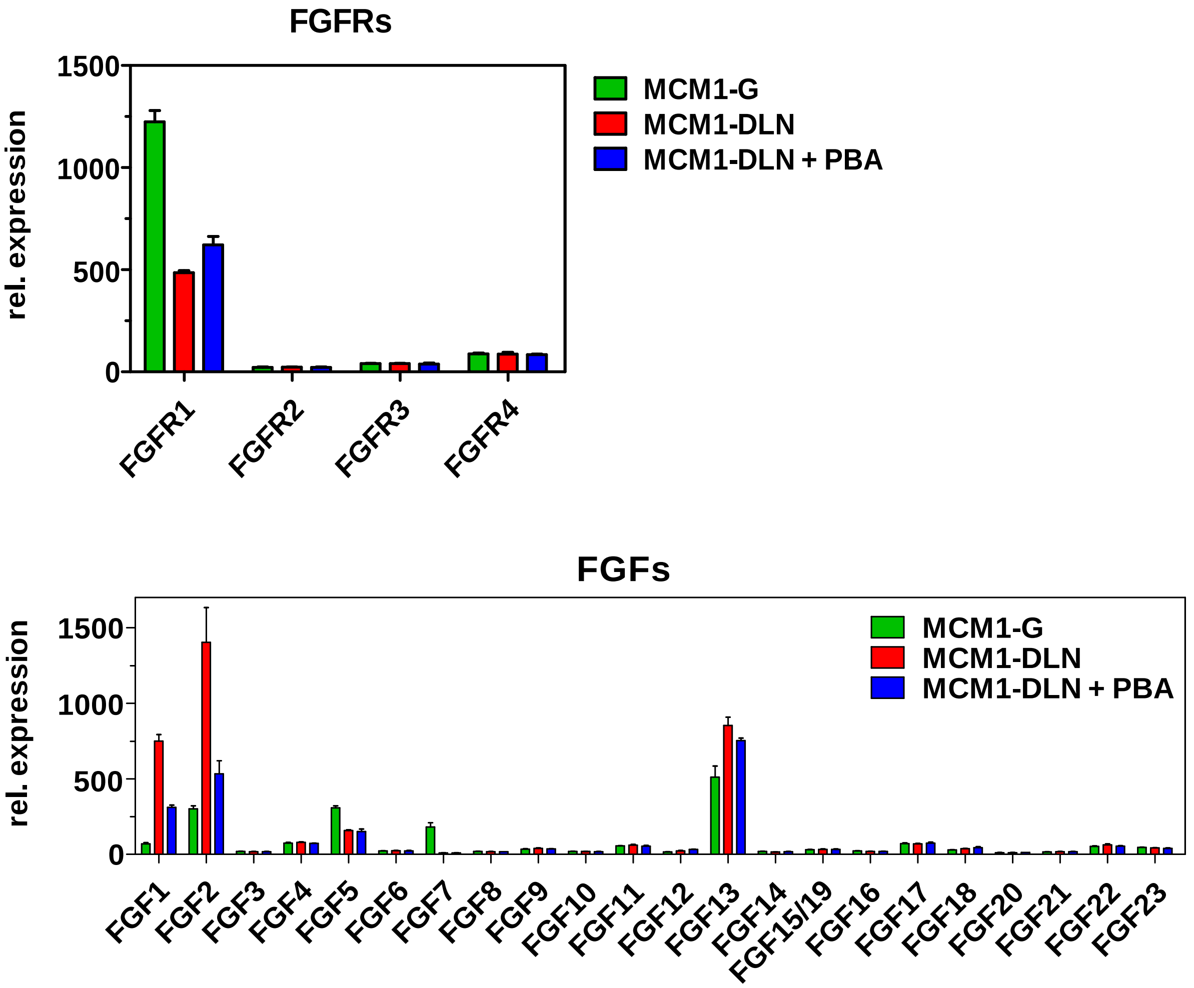

Supplementary Figure S3. Microarray expression data of FGFs and the FGF receptors in non-metastatic, metastatic and metastatic 4-PBA treated melanoma cell lines. The fibroblast growth factor-receptor 1 (FGFR1) is highly expressed in non-metastatic MCM1G, metastatic MCM1DLN and 4-PBA treated MCM1DLN cells, compared to low expression of the FGFR2, FGFR3 and FGFR4. Expression pattern of Fibroblast growth factors (FGFs) in melanoma cells: FGF1, FGF2 and FGF13 are the most highly expressed growth factors in metastatic MCM1DLN melanoma cells. Analyses were performed in quadruplicates.

Supplementary Figure S4.

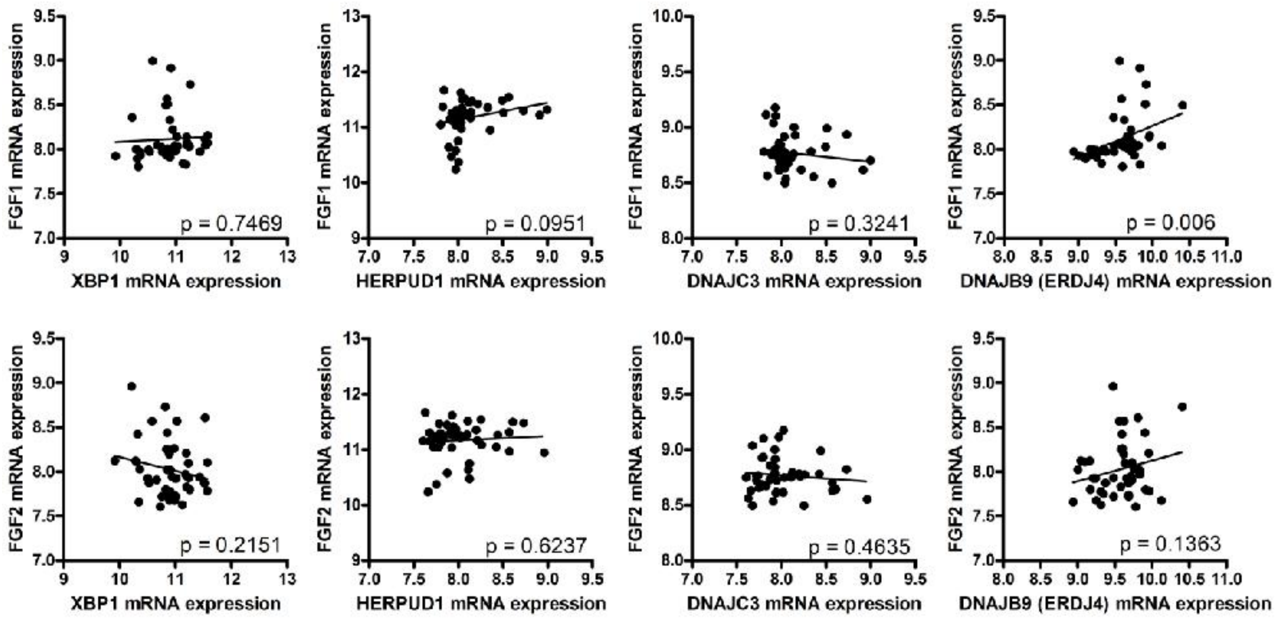

Supplementary Figure S4. Correlation of FGF1 and FGF2 with down-stream targets of the IRE1 pathway. No significant correlation of FGF1 with XBP1, HERPUD1, DNAJC3 and DNAJB9 was found. In line, no significant correlation of FGF2 with the abovementioned down-stream targets of the IRE1 pathway was observed. Data were retrieved from microarray expression experiments of human patient samples[1].

Supplementary Figure S5.

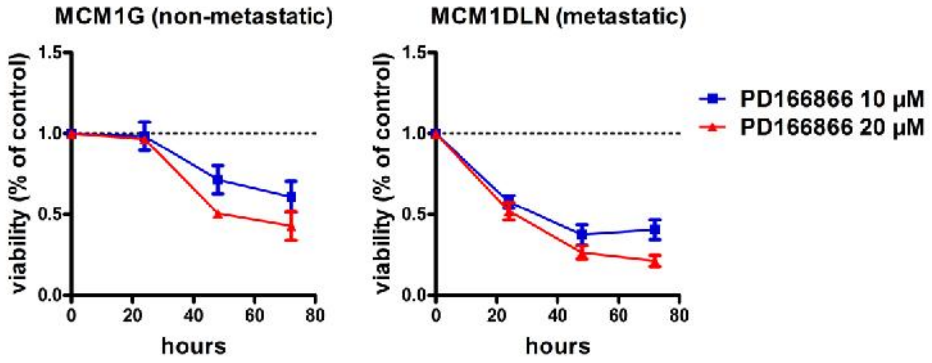

Supplementary Figure S5. Viability of non-metastatic MCM1G and metastatic MCM1DLN cells upon FGFR1 inhibition is reduced in a time dependent manner. Viability assay of 10 µM and 20 µM PD166866 treated MCM1G and MCM1DLN cells in MIM for the indicated time points (n=3).

Supplementary Figure S6.

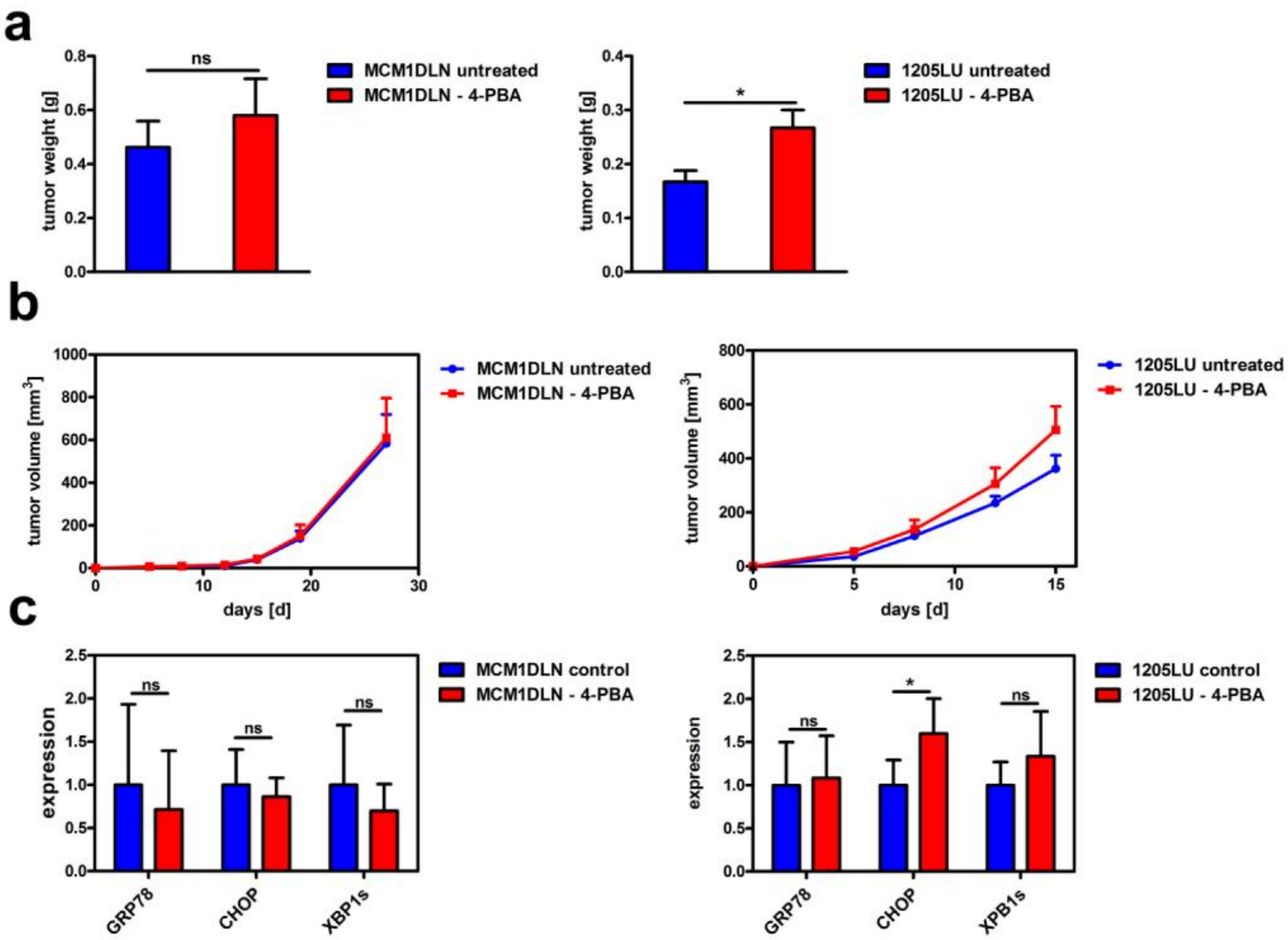

Supplementary Figure S6. Effects of 4-PBA on tumour progression *in-vivo*. (Fig. S6) 4-PBA does not reduce tumour weight (a), tumour volume (b) or UPR activity (c) in SCID mice xenografted with MCM1DLN or 1205Lu cells. No conspicuous effect of 4-PBA treatment on lung- or lymph node-metastasis was apparent (not shown).

Supplementary Figure S7.

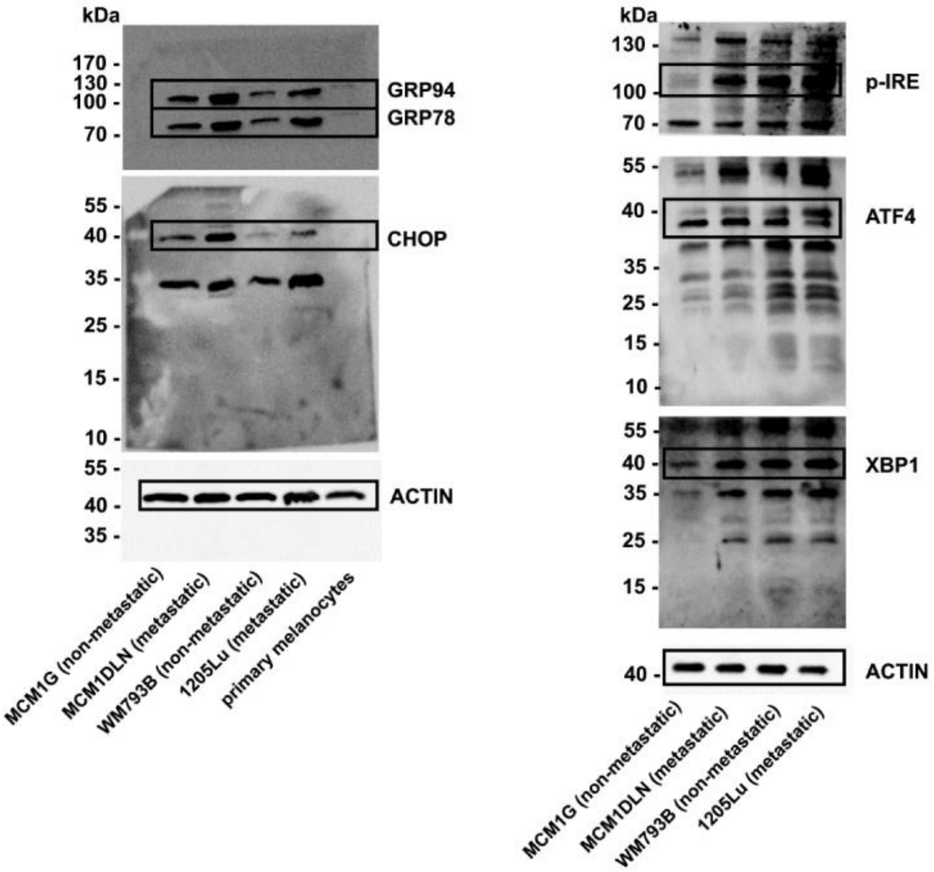

Supplementary Figure S7. Down-stream signalling of the unfolded protein response (UPR) is enhanced in metastatic melanoma cell lines. This image shows the original blot of the cropped images depicted in the main manuscript in Fig. 1b.

Supplementary Figure S8.

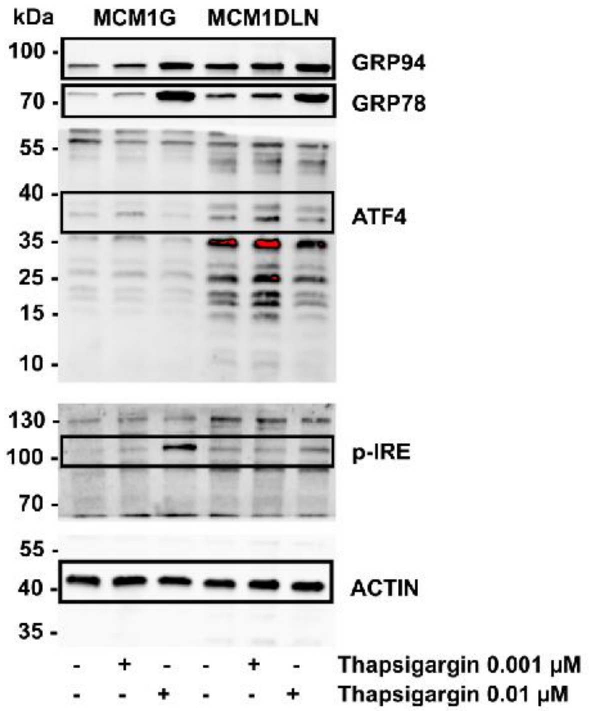

Supplementary Figure S8. Non-metastatic melanoma cells are more sensitive to acute ER stress induction. This image shows the original blot of the cropped images depicted in the main manuscript in Fig. 2d.

Supplementary Figure S9.

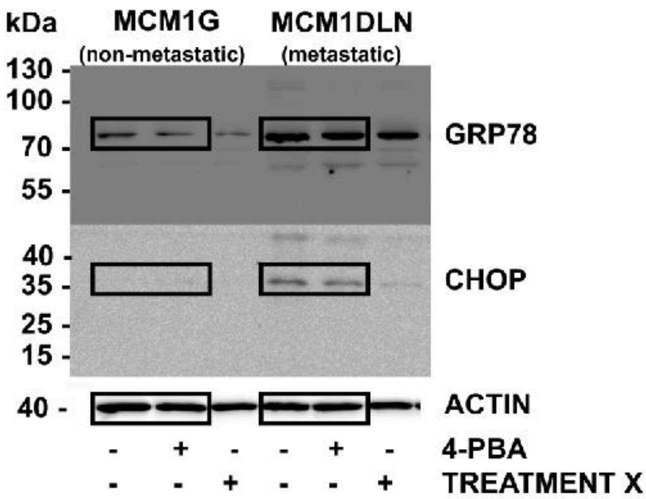

Supplementary Figure S9. Antagonization of the UPR using the chemical chaperone 4-PBA. This image shows the original blot of the cropped images depicted in the main manuscript in Fig. 4d.

Supplementary Figure S10.

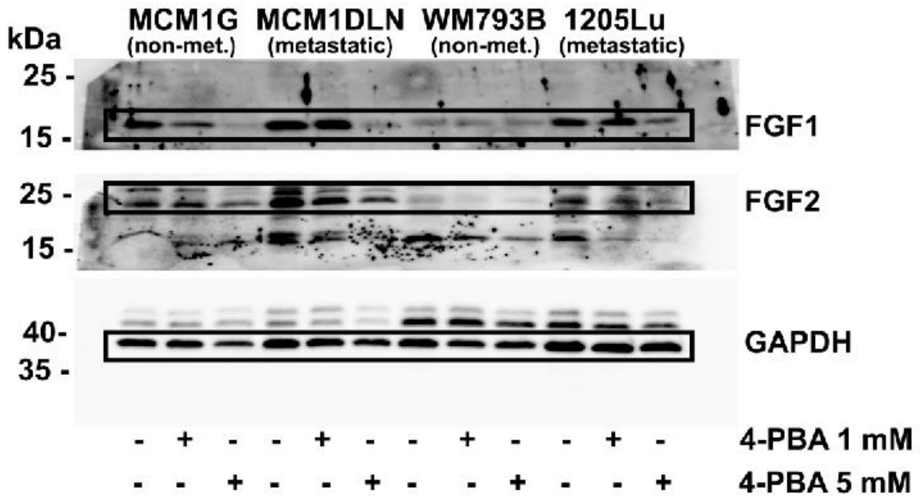

Supplementary Figure S10. FGF1 and FGF2 expression is diminished by 4-PBA in metastatic melanoma cells. This image shows the original blot of the cropped images depicted in the main manuscript in Fig. 5c. The bands above the GAPDH band result from a previous incubation with another antibody.

Supplementary References

1 Bogunovic D, O'Neill DW, Belitskaya-Levy I, Vacic V, Yu YL, Adams S, Darvishian F, Berman R, Shapiro R, Pavlick AC, Lonardi S, Zavadil J, et al. Immune profile and mitotic index of metastatic melanoma lesions enhance clinical staging in predicting patient survival. Proc Natl Acad Sci U S A 2009;106:20429-34.

2 Talantov D, Mazumder A, Yu JX, Briggs T, Jiang Y, Backus J, Atkins D, Wang Y. Novel genes associated with malignant melanoma but not benign melanocytic lesions. Clin Cancer Res 2005;11:7234-42.
